# Preference of saliva over other body fluids as samples for clinical and laboratory investigations among healthcare workers in Ibadan, Nigeria

**DOI:** 10.11604/pamj.2019.34.191.18738

**Published:** 2019-12-11

**Authors:** Taye Jemilat Lasisi, Folake Barakat Lawal

**Affiliations:** 1 Department of Physiology, University of Ibadan, Ibadan, Nigeria; 2 Department of Oral Pathology, University of Ibadan and University College Hospital, Ibadan, Nigeria; 3 Department of Periodontology & Community Dentistry, University of Ibadan and University College Hospital, Ibadan, Nigeria

**Keywords:** Clinical diagnosis, clinicians, laboratory tests, saliva, scientists

## Abstract

**Introduction:**

The study aimed to assess the knowledge and practices of clinicians and laboratory scientists on the use of saliva for clinical or laboratory tests.

**Methods:**

This was a cross-sectional survey of health care workers (100 clinicians and 62 laboratory scientists) closely involved with specimen collection for clinical and laboratory investigation at two health facilities (secondary and tertiary) in Nigeria. Information was obtained from participants using pretested structured questionnaires. Data were analyzed with SPSS and level of significance set at p < 5%.

**Results:**

The mean age of the study participants was 34.1 (±6.6) years. The majority (95.7%) knew saliva could be used for clinical/laboratory test. A higher proportion of laboratory scientists knew saliva could be used in diagnosing HIV (59.2%), oral diseases (88.7%), oro-facial tumors (64.4%) and genetic testing (94.5%) compared to (41%), (80%), (40%) and (80%), of clinicians respectively (p < 0.05). More clinicians (85%) indicated saliva as a good specimen for diagnosing systemic diseases compared with scientists (63%), p < 0.001. Saliva was the most comfortable/convenient body fluid to obtain from patients with more clinicians (80%) mentioning this than scientists (49.1%), p < 0.001. Twenty-six clinicians had used saliva for disease diagnosis (64%), treatment monitoring (28%) or research purposes (8%). Saliva sampling for research purposes was more prevalent among clinicians (p = 0.004).

**Conclusion:**

The majority of the health care workers knew the various uses and advantages of saliva as a specimen for clinical and laboratory investigation. However, few indicated previous use for clinical and laboratory investigation especially in the area of research.

## Introduction

Prompt disease diagnosis is not only relevant to reduce disease severity and prevent complications, but also crucial to achieve good success rate of therapy [1]. A great need exists for convenient and accurate point-of-care diagnostic tools that can be used in a non-invasive manner. This is of particular relevance in the developing world, where many health risks and illnesses remain poorly defined [2]. Saliva has been studied extensively as a potential diagnostic tool over the last decade due to its ease and non-invasive accessibility along with its abundance of biomarkers, such as genetic material and proteins [3-7]. Clinical practice and research rely on the collection of different body fluids including blood, saliva and urine to answer important questions about patients' health and risk status. In addition, federal and industrial funding sources have been used to develop saliva-based diagnostic tests [2, 3] and considerable progress has been made to elucidate the applicability of human saliva for disease diagnostics and monitoring [8-11]. However, its use for clinical or laboratory tests is still subject to the awareness as well as the acceptability of the concerned population especially the healthcare providers. No previous study on the knowledge and practices of healthcare workers on the use of saliva and other body fluids (blood and urine) for clinical testing has been documented from our environment. In addition, there is need for data to support the use of saliva over other traditional diagnostic fluids for clinical testing. The findings from this study may indicate health care workers' level of awareness of and receptivity towards saliva-based tests and probably health care workers based evidence in support of the reported ease of collection and point of care applicability of saliva testing. Thus, this study aimed at assessing the knowledge and practices of health care workers on the use of saliva for clinical and laboratory tests.

## Methods

This study received ethical approval from the Institution's Ethics Committee with approval number UI/UCH/EC/13/0099. It was a descriptive cross-sectional survey carried out at two health care facilities (one tertiary and the other a secondary health care facility) in Ibadan, Oyo State, Nigeria. All consenting clinicians and medical laboratory scientists available at the two health facilities during the period of the study were included. A convenience sampling technique was used to recruit the study participants at the two health care facilities. Information on biodata of the participants and their views on the use of saliva in clinical and laboratory testing were collected through structured questionnaires. The questions on the biodata of the participants were on their age, gender, profession and place of practice. The questionnaire assessed knowledge and practices of the participants on the use of saliva in clinical and laboratory testing, sources of this knowledge, the diseases that it can be used to investigate and diagnose, their preferred choice of sample collection among saliva and other body fluids, perceived advantages of saliva over other body fluids and previous saliva sampling for disease diagnosis and monitoring as well as research purposes. The questionnaire was self-administered and it was pretested among 20 clinicians and 10 laboratory scientists in the two facilities who were excluded from the main study to validate the questionnaire and determine the ease of answering the questions as well as the comprehensiveness. Prior to administration of the questionnaire, the purpose of the study was explained and only consenting health care workers were recruited for the study. Interns as well as doctors or scientists who were not available at the time of study were excluded. Data obtained was subjected to statistical analyses using SPSS version 23. Categorical data were summarized with frequencies and percentages and compared with chi square while quantitative data were summarized as means ± standard deviations (SD). For the purpose of bivariate analysis, age was dichotomized using the mean age. The p value for statistical significance was at < 0.05.

## Results

The mean age of the study participants was 34.1 (± 6.6) years and 56.2% were females. The majority (155, 95.7%) knew saliva could be used for clinical or laboratory test, with professional training (109, 70.3%) being the major source of knowledge (Table 1). Many (106, 68.4%) knew disease diagnosis as the main use of saliva. A higher proportion of scientists knew saliva could be used in diagnosing oral diseases (47, 88.7%), orofacial tumors (29, 64.4%) and for genetic testing (52, 94.5%), HIV (29, 59.2%), compared to clinicians (p < 0.05) (Table 2). However, more clinicians (85.0%) knew it as a good specimen for diagnosing systemic diseases compared with scientists (63.0%), p < 0.001 (Table 2). The advantages of saliva over other body fluid specimen, which the study participants strongly agreed or agreed with included; ease of collection (94.9%), elimination of fear of prick (92.5%) lower cost of sample collection (79.8%), reduced risk of infection (78.6%) and requirement of little or no skills for collection (75.1%). A higher proportion of clinicians (49%) indicated that saliva collection required no special skill compared to scientists (24.5%), p = 0.002. Saliva was the most preferred specimen to collect from patients by clinicians (44.0%), while blood specimen was preferred by scientists (46.4%). However, blood samples were the most preferred specimen to collect compared with other body fluids (Table 3). Of all the specimen, saliva was the most comfortable and convenient to obtain from patients and more clinicians (80%) mentioned this than scientists (49.1%), p < 0.001. In rating the level of convenience in specimen collection by the health care workers; scientists believed that blood samples and urine specimens were more convenient to collect than saliva samples (Table 3). Methods used by clinicians to collect saliva sample included spitting into specimen bottles (68.0%), use of cotton wool rolls (24.0%) and mechanical collector (8.0%). The venue of collection were clinics (88.0%) and laboratories (12.0%). The saliva samples collected by clinicians from patients were used for disease diagnosis (64.0%), research (28.0%) and treatment monitoring (8.0%). Saliva collection as samples for research purposes was practiced more often by clinicians (25.0%) than scientists (2.8%), p = 0.004. Diseases and conditions that necessitated collection of saliva specimen from patients and use for investigations included; oral diseases (32.0%), systemic diseases (28.0%), HIV (12.0%), genetic testing (8.0%) and others (20.0%) like tuberculosis, monitoring of urea levels, respiratory tract infections, oral microbial isolation and acid phosphatase analysis.

**Table 1:** Sources of knowledge of saliva as sample for investigation

Variable	Clinicians n (%)	Scientists n (%)	Total n (%)	X^2^	p value
**Knowledge of saliva as a specimen for investigation**					
Yes	95 (95.0)	60 (96.8)	155 (95.7)	2.811	0.245
No	0 (0.0)	1 (1.6)	1 (0.6)		
Don’t know	5 (5.0)	1 (1.6)	6 (3.7)		
Total	100 (100.0)	62 (100.0)	162 (100.0)		
**Source of knowledge**					
Professional training	69 (72.6)	40 (66.7)	109 (70.3)	6.565	0.161
Media (mass and social)	7 (7.4)	2 (3.3)	9 (5.8)		
Conferences	7 (7.4)	3 (5.0)	10 (6.5)		
Journals	12 (12.6)	13 (21.7)	25 (16.1)		
Others (friends, colleagues)	0 (0.0)	2 (3.3)	2 (1.3)		
**Total**	95 (100.0)	60 (100.0)	155 (100.0)		

**Table 2:** Knowledge of diseases that saliva can be used to diagnose among health care workers

Disease	Clinicians n (%)	Scientists n (%)	Total n (%)	X ^2^	P value
**Oral diseases**					
Yes	80 (80.0)	47 (88.7)	127 (83.0)	7.479	0.024^*^
No	1 (1.0)	3 (5.7)	4 (2.6)		
Don’t know	19 (19.0)	3 (5.7)	22 (14.4)		
Total	100 (100.0)	53 (100.0)	153 (100.0)		
**Systemic diseases**					
Yes	85 (85.0)	29 (63.0)	114 (78.1)	16.297	< 0.001^*^
No	1 (1.0)	8 (17.4)	9 (6.2)		
Don’t know	14 (14.0)	9 (19.6)	23 (15.8)		
Total	100 (100.0)	46 (100.0)	146 (100.0)		
**Orofacial tumors**					
Yes	40 (40.0)	29 (64.4)	69 (47.6)	7.955	0.019^*^
No	13 (13.0)	2 (4.4)	15 (10.3)		
Don’t know	47 (47.0)	14 (31.1)	81 (42.1)		
Total	100 (100.0)	45 (100.0)	145 (100.0)		
**Genetic testing**					
Yes	80 (80.0)	52 (94.5)	132 (85.2)	6.104	0.047^*^
No	2 (2.0)	0 (0.0)	2 (1.3)		
Don’t know	18 (18.0)	3 (5.5)	21 (13.5)		
Total	100 (100.0)	55 (100.0)	155 (100.0)		
**HIV**					
Yes	41 (41.0)	29 (59.2)	70 (47.0)	13.225	0.001^*^
No	29 (29.0)	18 (36.7)	47 (31. 5)		
Don’t know	30 (30.0)	2 (4.1)	32 (21.5)		
Total	100 (100.0)	49 (100.0)	149 (100.0)		

* Statistically significant

**Table 3:** Preference for body fluids samples for investigation and ratings of convenience of their collection from patients among health care workers

Variables	Clinicians n (%)	Scientists n (%)	Total n (%)	X^2^	P value
**Preferred sample to collect for investigation**					
Saliva	44 (44.0)	12 (21.4)	56 (35.9)	12.693	0.005*
Blood	43 (43.0)	26 (46.4)	69 (44.3)		
Urine	11 (11.0)	17 (30.4)	28 (17.9)		
Indifferent	2 (2.0)	1(1.8)	3 (1.9)		
**Most convenient and comfortable sample to collect for investigation**					
Saliva	80 (80.0)	27 (49.1)	107 (69.0)	28.962	< 0.001*
Blood	4 (4.0)	20 (36.4)	24 (15.5)		
Urine	10 (10.0)	5 (9.1)	15 (9.7)		
Indifferent	6 (6.0)	3 (5.5)	9 (5.8)		
**Rating of convenience in collecting saliva sample from patients**					
Much convenient	89 (89.0)	41 (74.2)	130 (83.9)	14.456	0.002*
Less convenient	6 (6.0)	13 (23.6)	19 (12.3)		
Not convenient	0 (0.0)	1 (1.8)	1 (0.6)		
Indifferent	5 (5.0)	0 (0.0)	5 (3.2)		
**Rating of convenience in collecting blood sample from patients**					
Much convenient	16 (16.0)	35 (63.6)	31 (32.9)	40.493	<0.001*
Less convenient	52 (52.0)	18 (32.7)	70 (45.2)		
Not convenient	27 (27.0)	2 (3.6)	29 (18.7)		
Indifferent	5 (5.0)	0 (0.0)	5 (3.2)		
**Rating of convenience in collecting urine sample from patients**					
Much convenient	32 (32.0)	28 (51.9)	60 (39.0)	7.637	0.054
Less convenient	52 (52.0)	22 (40.7)	74 (48.1)		
Not convenient	11 (11.0)	4 (7.4)	15 (9.7)		
Indifferent	5 (5.0)	0 (0.0)	5 (3.2)		

* Statistically significant

## Discussion

To the best of our knowledge this study is the first to report the knowledge and attitude of clinicians and laboratory scientists on the use of saliva for diagnosis. Majority of the participants in this study knew that saliva could be used for clinical or laboratory test with professional training as the major source of knowledge. Similarly, many of the respondents indicated disease diagnosis as the main use of saliva. Conventionally, diseases are diagnosed based on patient reported symptoms, medical history obtained by a physician or other medical professional and biochemical analysis of blood and/or urine samples. The patient's samples are usually sent to the diagnostic laboratory for analysis of the levels of different markers such as ions, antibodies, proteins, hormones, cytokines, and a variety of disease-specific biomarkers. There has been increasing knowledge about the use of saliva for clinical and laboratory diagnosis as observed in this study and previous reports [12-15]. This increased awareness is possibly the outcome of numerous reports on the use of saliva for the diagnosis as well as monitoring of oral and systemic diseases [16-18]. More importantly, if it is possible to obtain similar or identical information with saliva sample that is easy to collect and that does not require invasive procedures, the need for a blood draw would become unnecessary. This is particularly important in a number of populations and situations, which include handling pediatric and geriatric patients, or when access to health care is limited in remote geographic areas or rural communities where phlebotomists are unavailable. Similar to our finding, a survey reported that dentists believed that screening for medical conditions (in the dental clinics) is important and they indicated willingness to participate when the sample is saliva, as opposed to a finger stick [19]. Many advantages have been attributed to the use of saliva samples (for disease diagnosis) over other body fluids in the literature [1, 2, 9-13]. The advantages of saliva over other body fluid specimen include; ease of collection, elimination of fear of prick, lower cost of sample collection, reduced risk of infection and requirement of little or no skills for collection. Finding from this study indicated that majority of the respondents also strongly agreed or agreed with these advantages of using saliva for disease diagnosis over other body fluids (blood and urine). The finding of a higher proportion of clinicians indicating that saliva collection required no special skill compared to scientists could be explained by the nature of their practice. Clinicians are more often involved in taking samples from patients whereas the scientists usually receive the samples (after collection) for the laboratory analysis. In addition, the finding that most of the respondents indicated that samples are collected in the clinics as against the laboratories could also explain why a higher proportion of clinicians indicated that saliva collection required no special skill compared to scientists.

Of the various methods of saliva collection, spitting into specimen bottles was the most common method used by the clinician respondents in this study, whereas use of mechanical collector was the least method indicated. This finding agrees with many reports [6, 7, 16, 18] that used saliva samples and this could be attributed to the fact that the spitting method is more convenient and less expensive compared to the other methods. For example, use of mechanical collector or device requires a selection of commercially available devices for the collection and some of these devices are not readily available in the developing countries. Also, using this method may result in spending more money for the procurement of the materials, which might end up making it more expensive. Of all the types of specimen, saliva was chosen as the most comfortable and convenient to obtain from patients and more clinicians mentioned this than scientists. However, blood samples were the most preferred specimen to collect compared with other body fluids (blood and urine). Saliva was the most preferred specimen to collect from patients by clinicians, while blood specimen was preferred by scientists. In addition, in rating the level of convenience in specimen collection by the health care workers; scientists believed that blood samples and urine specimens were more convenient to collect than saliva samples. This finding may be related to the non-familiarity of the scientists with saliva sampling due to the fact that only a few of the scientists also indicated that they have collected saliva samples before. The saliva samples collected by clinicians from patients were used mostly for disease diagnosis rather than for research and treatment monitoring. In addition, saliva collection as samples for research purposes was practiced more often by clinicians than scientists. These findings indicate the necessity for the increased use of saliva samples in research activities by clinicians and scientists, more so that saliva has many constituents that can be explored. Importantly, the advent of modern technology and current development of diagnostic biomarkers via proteomic and genomic approaches has broadened the use of saliva for various analyses that can be used in making clinical decisions and predicting treatment outcomes [2, 4, 5]. Along with these developments are advancements to overcome barriers such as technological problems related to achieving high sensitivity, high specificity, miniaturization, high throughput (assay of a large number of samples concurrently), automation, portability, low cost, high functionality and speed [2]. Overcoming these barriers will encourage widespread implementation of salivary diagnostics by the clinicians and scientists.

Diseases and conditions that saliva specimen had been collected from patients and used to investigate included oral diseases (32.0%), systemic diseases (28.0%), HIV (12.0%), genetic testing (8.0%) and others (20.0%) like tuberculosis, urea monitoring, respiratory tract infections, oral microbial isolation and acid phosphatase analysis. These findings indicate that there is still a large vacuum in the use of saliva for important diagnostic uses. This may be attributed to the lack of facilities and appropriate funding for research activities in our environment. Also, the low patronage of use of saliva for disease diagnosis as indicated in this survey may be explained by lack of correlation between plasma and saliva levels of some parameters. For example, the second major use of saliva samples is for the quantitation of steroid hormone levels. Assays have shown consistent accurate detection of hormones such as cortisol, estriol, estrogen and testosterone [20-23]. However, salivary levels do not correlate well with serum levels in the case of conjugated steroid hormones [24]. These observations raise the general issue of a qualitative versus quantitative assay for biomarkers in saliva. When qualitative (such as absent versus present or yes versus no) results are needed, as in the case of malarial parasite, pregnancy, bacterial and viral infections, saliva sampling will mostly be useful. When a quantitative result is needed however, for example when evaluating levels or concentrations of parameters such as glucose, DHEA-S, creatinine, urea etc, the determination of the saliva/plasma ratio is necessary. The closer the saliva/plasma ratio to 1 (e.g. as for ethyl alcohol and unconjugated steroid hormones), the more feasible the quantitative salivary testing for the parameter and if not, then a quantitative salivary-based assay of that parameter may not be suitable for that biomarker [24]. Another challenge in making salivary diagnostics a clinical reality is establishing the scientific foundation and clinical validations needed to position it as a highly accurate and feasible technology that can achieve definitive point of care assessment of health and disease status. Important in this aspect, is the establishment of scientific and diagnostic biomarkers in saliva and the development of robust, simple-to-use biosensor technologies for reliable and valid clinical applications.

## Conclusion

This survey has indicated that the majority of the health care workers knew the various uses and advantages of saliva as a specimen for clinical and laboratory investigation. However, few indicated previous use for clinical as well as laboratory investigation especially in the area of research. The ability to use saliva to monitor a patient's health and disease states is a highly desirable goal for health promotion and health care research. However, additional education and practical implementation strategies are necessary to address perceived barriers.

### What is known about this topic

- Obtaining body fluids for research in the community or in clinic settings is quite challenging in Nigeria;
- Patients and research subjects often do not want to give body fluids such as blood because difficulty in obtaining the samples.

### What this study adds

- Saliva is the most convenient and comfortable body fluid to obtain from patients;
- Clinicians are more supportive of saliva sampling for research than laboratory scientists.

## Competing interests

The authors declare no competing interests.
